# SYNTAX score II predicts long-term mortality in patients with one- or two-vessel disease

**DOI:** 10.1371/journal.pone.0200076

**Published:** 2018-07-02

**Authors:** Maxime M. Vroegindewey, Anne-Sophie Schuurman, Rohit M. Oemrawsingh, Robert-Jan van Geuns, Isabella Kardys, Jurgen Ligthart, Joost Daemen, Eric Boersma, Patrick W. Serruys, K. Martijn Akkerhuis

**Affiliations:** 1 Department of Cardiology, Thoraxcenter, Erasmus MC, Rotterdam, The Netherlands; 2 Department of Cardiology, Amphia Ziekenhuis, Breda, The Netherlands; 3 Department of Cardiology, Imperial College, London, United Kingdom; University of Tampere, FINLAND

## Abstract

**Objective:**

SYNTAX score II (SSII) is a long-term mortality prediction model to guide the decision making of the heart-team between coronary artery bypass grafting or percutaneous coronary intervention (PCI) in patients with left main or three-vessel coronary artery disease. This study aims to investigate the long-term predictive value of SSII for all-cause mortality in patients with one- or two-vessel disease undergoing PCI.

**Methods:**

A total of 628 patients (76% men, mean age: 61±10 years) undergoing PCI due to stable angina pectoris (43%) or acute coronary syndrome (57%), included between January 2008 and June 2013, were eligible for the current study. SSII was calculated using the original SYNTAX score website (www.syntaxscore.com). Cox regression analysis was used to assess the association between continuous SSII and long-term all-cause mortality. The area under the receiver-operating characteristic curve was used to assess the performance of SSII.

**Results:**

SSII ranged from 6.6 to 58.2 (median: 20.4, interquartile range: 16.1–26.8). In multivariable analysis, SSII proved to be an independent significant predictor for 4.5-year mortality (hazard ratio per point increase: 1.10; 95% confidence interval: 1.07–1.13; p<0.001). In terms of discrimination, SSII had a concordance index of 0.77.

**Conclusion:**

In addition to its established value in patients with left main and three-vessel disease, SSII may also predict long-term mortality in PCI-treated patients with one- or two-vessel disease.

## Introduction

The SYNTAX score II (SSII) has been established as a long-term mortality prediction model to guide the decision making of the heart-team between coronary artery bypass grafting (CABG) or percutaneous coronary intervention (PCI) in patients with complex coronary artery disease (CAD).[1] It combines the original anatomical-based SYNTAX score, which grades the complexity of CAD in all coronary arteries, with the clinical baseline variables that have shown to be important predictors of 4-year all-cause mortality in the SYNTAX trial.[1]

SSII has been validated in large patient cohorts with left main or three-vessel disease.[1–3] However, the predictive performance of SSII on long-term mortality in patients with less complex CAD is currently unknown.

This study aims to investigate the long-term predictive value of SSII for all-cause mortality in patients with one- or two-vessel disease undergoing PCI.

## Methods

### Study design and population

This study combines the populations of The European Collaborative Project on Inflammation and Vascular Wall Remodeling in Atherosclerosis-intravascular ultrasound (ATHEROREMO-IVUS) study and the Integrated Biomarker and Imaging Study-3 (IBIS-3).[4, 5] Study designs and methods of ATHEROREMO-IVUS and IBIS-3 have been described in detail elsewhere.[4, 5] Baseline study procedures and inclusion criteria were similar and both studies were conducted at the Erasmus Medical Center, Rotterdam, The Netherlands. In brief, patients undergoing diagnostic coronary angiography (CAG) or PCI for an acute coronary syndrome (ACS) or stable angina pectoris (SAP) were included. During CAG, invasive imaging was performed in one non-culprit coronary artery segment. Subsequently, patients were followed-up on adverse cardiovascular events. Patient care was left at the discretion of the physician. During the first year after the index procedure, as per protocol, patients included in IBIS-3 received high dose rosuvastatin.

The medical ethics committee of the Erasmus MC approved both the ATHEROREMO-IVUS and IBIS-3 study. Both studies were performed in accordance with the declaration of Helsinki. All patients provided written informed consent for their participation in these studies, and compliance with the study protocols, including long-term follow-up. The ATHEROREMO-IVUS study is registered in ClinicalTrials.gov, number NCT01789411, and the IBIS-3 study is registered in The Netherlands trial register, number NTR2872.

### SYNTAX score II

The anatomical-based SYNTAX score was determined (pre-PCI) for every coronary angiogram taken at study entry, by a trained analyst blinded for patient characteristics and outcome using the SYNTAX Score Calculator (www.syntaxscore.com). The calculation of the anatomical-based SYNTAX score has been described in detail previously.[6] In brief, the complete coronary tree is divided in 16 segments and assessed for lesions producing 50% or more luminal obstruction. Every segment has a pre-specified corresponding weighing factor which, in case of a significant lesion, is added to the SYNTAX score by the SYNTAX Score Calculator. Moreover, other variables (i.e. calcification or lesion length) that reflect the complexity of a patient’s CAD and, thus, the complexity of treatment are assessed and taken into account in the SYNTAX score. Eventually, the SYNTAX score is composed of these total points summed, and reflects the complexity of a patient’s CAD. As previously applied in other all-comers and ST-segment elevation myocardial infarction (STEMI) populations, lesions caused by in-stent restenosis were treated as de novo lesions.[7–9] Total occlusions were scored as occlusions of unknown duration, as the analyst was blinded for all patient information.[10]

Subsequently, data on the baseline variables age, gender, creatinine clearance (CRCL), left ventricular ejection fraction (LVEF), peripheral vascular disease and chronic obstructive pulmonary disease was collected for the calculation of SSII. We used the original SYNTAX Score II Calculator (www.syntaxscore.com) to obtain all SSII values. The algorithm of the SSII calculation has been described in detail elsewhere.[1]

### Study endpoint

The primary endpoint was all-cause mortality. Vital status of the patients was obtained from municipal civil registries.

### Statistical analysis

The distribution of continuous variables was examined for normality with the Kolmogorov-Smirnov test. ANOVA or Kruskal-Wallis test were used for multiple group comparison of continuous variables. Categorical variables were compared using the Pearson Chi-square test.

Data for most of the variables used for the calculation of SSII were complete. However, creatinine, required for the calculation of CRCL, was available in 92.8% of the patients. LVEF was available in 72.0% of the patients and categorized as good (LVEF≥50%), moderate (LVEF 40–49%) and poor (LVEF<40%).[11] Because LVEF was reported qualitatively, a value of 50% was used for category good, 44.5% for category moderate and 35% for category poor for the calculation of SSII. Multiple imputation technique was used to impute the missing data of creatinine and LVEF. Ten imputed data sets were generated. Analyses were conducted for both the complete dataset as the imputed datasets, which showed similar results.

Long-term cumulative incidences of all-cause mortality, categorized by SSII in tertiles, were compared with the log-rank test. Cox regression analysis was used to assess the association between continuous SSII and long-term all-cause mortality. Patients that were lost to follow-up were censored at the date of last contact. Based on existing literature, variables known to be associated with mortality and not part of the SSII (diabetes mellitus, hypertension, smoking, previous PCI and indication for coronary angiography) were entered in a multivariable Cox model. Since our study population also includes STEMI-patients and the use of SSII has been validated in stable patients, a subgroup analysis was performed in patients with SAP only, to compare the results of the total study population with the results found in patients with SAP only. In terms of discrimination, the area under the receiver-operating characteristic (ROC) curve was assessed. All statistical test were two-sided with a type I error level of 0.05. Analyses were performed with IBM SPSS Statistics version 21.0.

## Results

A total of 628 patients (76% men, mean age: 61 ±10 years) undergoing PCI due to SAP (43%) or ACS (57%), included between January 2008 and June 2013, were eligible for the current study (Table 1). SSII ranged from 6.6 to 58.2 (median: 20.4, IQR: 16.1–26.8). All-cause mortality occurred in 44 patients (7.0%) during a median follow-up of 4.5 (IQR: 3.4–4.9) years. Patients with a high SSII were older, had a higher prevalence of diabetes mellitus, hypertension, hypercholesterolemia and COPD, and more frequently had a history of renal insufficiency or heart failure than patients with a mid or low SSII.

**Table 1:** Baseline characteristics.

*Clinical characteristics*	SSII ≤17 (n = 209)	17< SSII ≤24 (n = 210)	SSII >24 (n = 209)	p value
Age—yrs, ± sd	52.9 ± 7.8	61.5 ± 8.1	69.0 ± 9.2	<0.001
Men, n (%)	204 (97.6)	163 (77.6)	109 (52.2)	<0.001
Diabetes mellitus, n (%)	28 (13.4)	40 (19.0)	49 (23.4)	0.051
Hypertension, n (%)	91 (34.5)	112 (53.3)	134 (64.1)	<0.001
Hypercholesterolemia, n (%)	96 (45.9)	120 (57.1)	127 (60.8)	0.025
Current smoking, n (%)	88 (42.3)	60 (28.6)	47 (22.5)	<0.001
Previous MI, n (%)	55 (26.3)	57 (27.1)	64 (30.6)	0.48
Previous PCI, n (%)	58 (27.8)	65 (31.0)	60 (28.7)	0.74
Previous CVA, n (%)	10 (4.8)	10 (4.8)	18 (8.6)	0.16
History of PAD, n (%)	0 (0.0)	0 (0.0)	46 (22.0)	<0.001
History of renal insufficiency, n (%)	6 (2.9)	4 (1.9)	20 (9.6)	<0.001
History of heart failure, n (%)	1 (0.5)	2 (1.0)	10 (4.8)	0.003
COPD, n (%)	1 (0.5)	9 (4.3)	23 (11.0)	<0.001
Serum creatinine—μmol/L, ± sd	77.3 ± 13.2	74.8 ± 17.3	84.6 ± 28.3	<0.001
Creatinine clearance—ml/min, ± sd	127.8 ± 31.8	111.9 ± 34.3	80.1 ± 27.8	<0.001
LVEF, n (%)				<0.001
Good LVEF ≥50%	189 (90.4)	156 (74.2)	136 (65.1)	
Moderate LVEF 40–49%	20 (9.6)	54 (25.8)	65 (31.1)	
Poor LVEF <40%	0 (0.0)	0 (0.0)	8 (3.8)	
*Angiographic characteristics*				
Indication for angiography, n (%)				0.16
Acute MI	77 (36.8)	64 (30.6)	51 (24.4)	
Unstable angina	58 (27.8)	57 (27.3)	61 (29.2)	
Stable angina	74 (35.4)	99 (47.4)	97 (46.4)	
Coronary artery disease, n (%)				0.007
1-vessel disease	146 (69.9)	118 (56.5)	115 (55.0)	
2-vessel disease	63 (30.1)	92 (43.5)	94 (45.0)	
Median SS [IQR]	5.0 [3.0–9.0]	9.0 [5.0–13.5]	9.0 [5.0–15.0]	<0.001

CI: confidence interval; COPD: Chronic obstructive pulmonary disease; CVA: Cerebrovascular accident; IQR: inter quartile range; LVEF: left ventricular ejection fraction; MI: Myocardial infarction; PAD: Peripheral artery disease; PCI: Percutaneous coronary intervention; sd: standard deviation; SS: SYNTAX score; SSII: SYNTAX score II; yrs: years.

**Fig 1:**
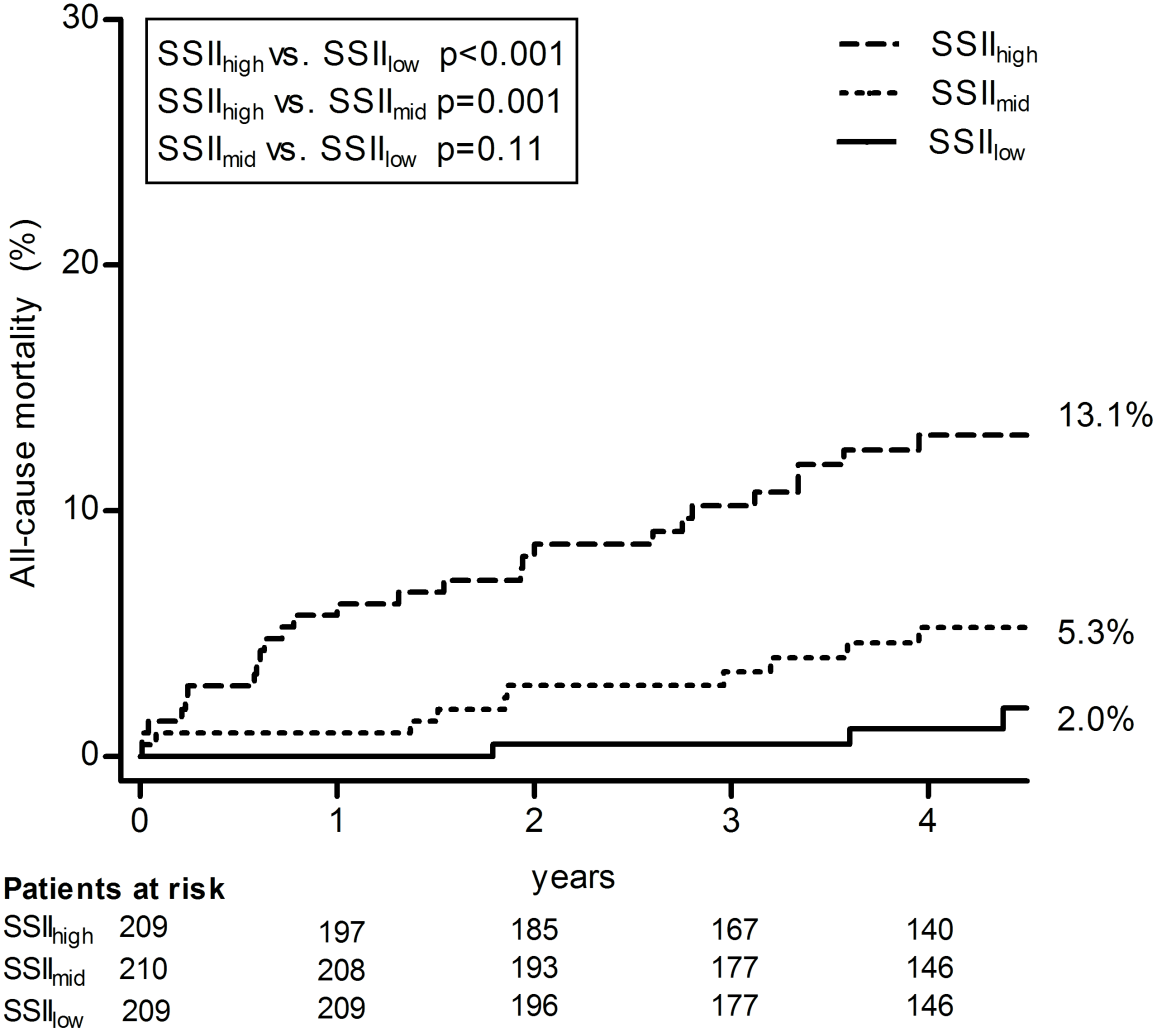
Cumulative incidence of all-cause mortality at 4.5 years. SSII is divided in tertiles with cut-off points 17 and 24 to compare the cumulative all-cause mortality proportions between patients with a low, mid or high SSII value. SSII: Syntax score II.

Cumulative incidence of all-cause mortality categorized by SSII in tertiles is shown in Fig 1. The long-term cumulative incidence of all-cause mortality of patients with a high SSII showed to be significantly higher than for patients with a mid or low SSII. No statistically significant difference was found between the cumulative incidence of all-cause mortality of patients with a mid versus low SSII value.

**Fig 2:**
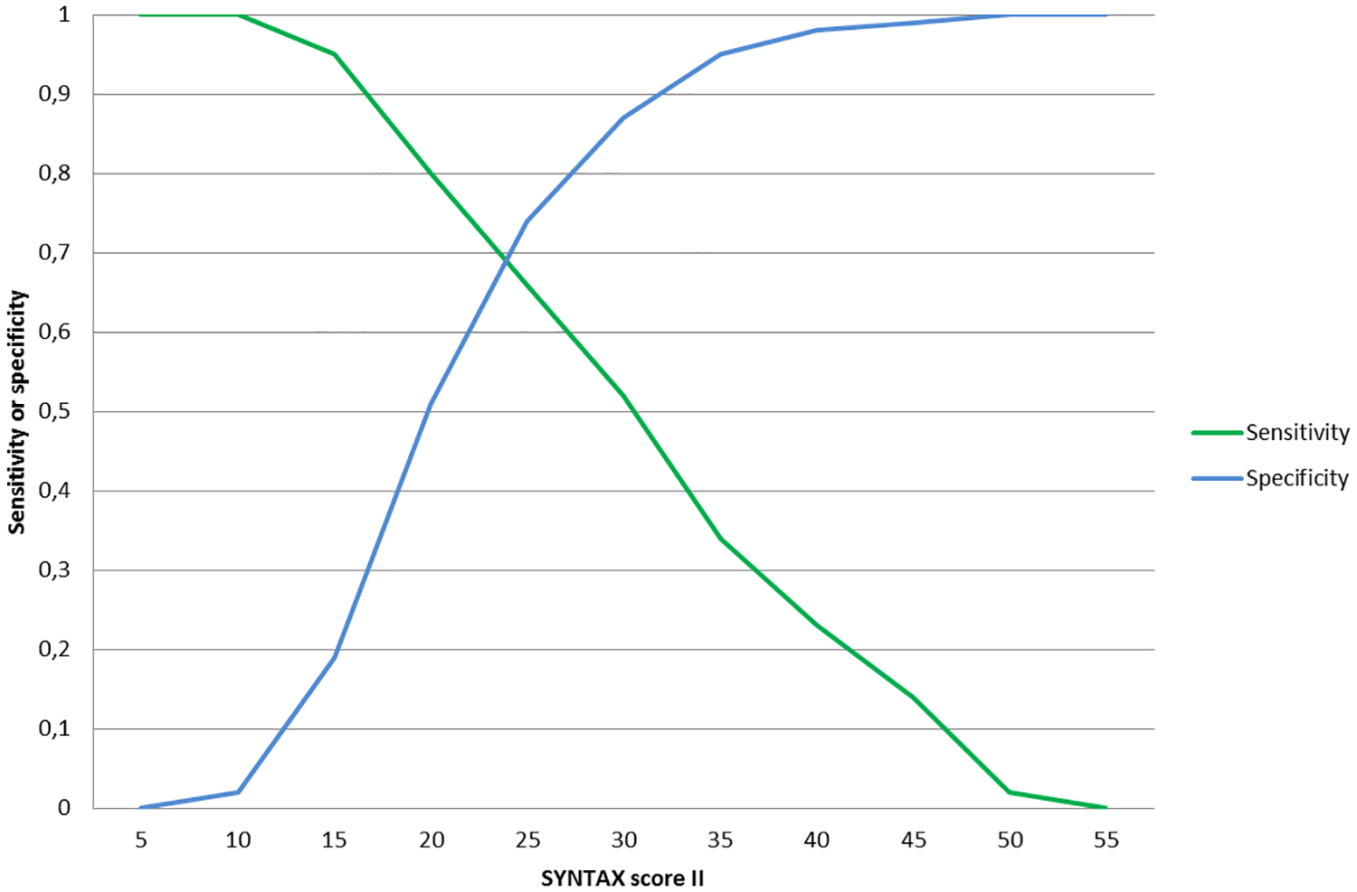
Sensitivity and specificity of SSII for the long-term prediction of all-cause mortality.

In the multivariable Cox model (Table 2), SSII proved to be an independent significant predictor for 4.5-year mortality (hazard ratio [HR] per point increase: 1.10; 95% confidence interval [CI]: 1.07–1.13). For SAP patients only, results were similar to the total study population (HR: 1.06; 95%CI: 1.07–1.11). In terms of discrimination, SSII had a concordance index (c-index) of 0.77 (95%CI: 0.69–0.84) (Fig 2).

**Table 2:** Prediction of long-term mortality.

*Total population (n = 628)*			*SAP patients only (n = 270)*		
	Unadjusted HR (95%CI)	p value		Unadjusted HR (95%CI)	p value
SSII	1.09 (1.07–1.12)	<0.001	SSII	1.05 (1.00–1.10)	0.050
	Adjusted HR (95%CI)			Adjusted HR (95%CI)	
SSII	1.10 (1.07–1.13)	<0.001	SSII	1.06 (1.07–1.11)	0.037
Smoking	1.01 (0.52–1.98)	0.97	Smoking	1.52 (0.48–4.82)	0.48
Diabetes mellitus	1.60 (0.79–3.24)	0.19	Diabetes mellitus	1.56 (0.52–4.62)	0.43
Hypertension	0.88 (0.46–1.68)	0.70	Hypertension	0.59 (0.21–1.71)	0.33
Previous PCI	1.09 (0.54–2.18)	0.82	Previous PCI	0.59 (0.20–1.75)	0.35
Indication for CAG, SAP	0.59 (0.30–1.15)	0.12			

SSII incorporates the anatomical Syntax score, age, gender, creatinine clearance, left ventricular ejection fraction, peripheral vascular disease and chronic obstructive pulmonary disease

CAG: coronary angiography; CI: confidence interval; HR: hazard ratio; PCI: percutaneous coronary intervention; SAP: stable angina pectoris; SSII: Syntax score II

## Discussion

In this study, we validated for the first time the use of SSII for prediction of long-term mortality in a large PCI-treated patient population with one- or two-vessel disease. SSII demonstrated to be an independent predictor for 4.5-year all-cause mortality in multivariable analysis. Moreover, in terms of discrimination, SSII had a c-index of 0.77 which is in line with the internally and externally validated c-indices of 0.73 and 0.72, respectively, of SSII in the SYNTAX trial for CABG or PCI-treated patients with left main or three-vessel disease.[1] It is also in line with the c-index (0.75) of SSII found in a study of patients with left main or three-vessel disease treated with only PCI.[3]

Although other known predictors of all-cause mortality in patients with CAD which are not part of SSII, such as diabetes mellitus, hypertension and prior PCI, were entered in the multivariable Cox model, SSII demonstrated to be the only significant predictor for 4.5-year all-cause mortality. Diabetes mellitus is a well-known predictor for adverse outcome in patients treated with PCI.[12] However, our findings imply that SSII incorporates enough relevant clinical prognostic variables to predict long-term all-cause mortality in patients with one- or two-vessel disease. Recently, the performance of SSII has been compared in diabetic patients versus non-diabetic patients with multi-vessel or left main disease undergoing PCI.[13] The SSII showed to have a good discriminative ability in both patient groups, independent of diabetic status. It may be hypothesized that other clinical variables incorporated in the SSII, such as CRCL, sufficiently reflect the influence of diabetes mellitus. In this respect, a previous study has demonstrated that kidney disease is of greater importance than diabetes mellitus for risk prediction of adverse outcome in patients with CAD.[14]

SSII has been developed for individual risk assessment using a continuous scale to overcome the limitations of categorized risk scores. Our study validates the use of SSII in patients with one- or two-vessel disease, demonstrating a similar discrimination as previously reported in left main or three-vessel disease.

### Limitations

In our study, the calculation of anatomical-based SYNTAX score for SSII included small vessels of at least 1.5mm and intermediate stenosis causing luminal obstruction of <70%, as instructed by the SYNTAX trial.[6] However, as recently observed in prospective registries, intermediate stenosis and small vessels <2.0 mm may not have additive predictive value for the prognosis of late mortality.[15–17] Hence, SSII calculated when only including severe stenosis of >70% in vessels of at least 2 mm, may even more accurately predict late mortality than currently observed in our study.

Further, the modest reproducibility of the anatomical-based SYNTAX score has to be acknowledged.[18] However, since our study population with one- or two-vessel disease had a relatively low angiographic burden, we expected a fair reproducibility of the anatomical-based SYNTAX score. To assess the reproducibility, a second experienced analyst repeated the anatomical-based SYNTAX score analysis in a representative random sample, blinded for patient information and previously scored SXscores. Cohen’s kappa was 0.91, which indicated a good interobserver agreement. Furthermore, since SSII is used in a continuous manner and incorporates both anatomical as well as clinical variables, SSII offers higher accuracy than the original anatomical-based SYNTAX score.[3]

In addition, our single-center study needs external validation. As expected, the median SSII score in our population was lower than in the original SSII report and further research is required to investigate the relation between the actual SSII and corresponding event rate in one- or two-vessel disease.

### Conclusion

This study validates the predictive performance of SSII in patients with one- or two- vessel disease indicating that, in addition to its known value in patients with left main or three-vessel disease, SSII may also offer accurate risk prediction in patients with less complex CAD.

## Supporting information

S1 Dataset Data file. (SAV)
